# Neurobiological substrates of altered states of consciousness induced by high ventilation breathwork accompanied by music

**DOI:** 10.1371/journal.pone.0329411

**Published:** 2025-08-27

**Authors:** Amy Amla Kartar, Toru Horinouchi, Balázs Örzsik, Brittany Anderson, Lottie Hall, Duncan Bailey, Sarah Samuel, Nati Beltran, Samira Bouyagoub, Chris Racey, Yoko Nagai, Iris Asllani, Hugo Critchley, Alessandro Colasanti

**Affiliations:** 1 Brighton and Sussex Medical School, Department of Clinical Neuroscience, University of Sussex, United Kingdom; 2 Department of Psychiatry and Neurology, Hokkaido University Hospital, Japan; 3 Radiology, Leiden University Medical Centre, Leiden, Netherlands; 4 University of Wisconsin School of Medicine & Public Health, Department of Psychiatry, University of Wisconsin–Madison, United States of America; 5 Sussex Partnership NHS Foundation Trust, Worthing, United Kingdom; Institut VEDECOM, FRANCE

## Abstract

The popularity of breathwork as a therapeutic tool for psychological distress is rapidly expanding. Breathwork practices that increase ventilatory rate or depth, facilitated by music, can evoke subjective experiential states analogous to altered states of consciousness (ASCs) evoked by psychedelic substances. These states include components such as euphoria, bliss, and perceptual differences. However, the neurobiological mechanisms underlying the profound subjective effects of high ventilation breathwork (HVB) remain largely unknown and unexplored. In this study, we investigated the neurobiological substrates of ASCs induced by HVB in experienced practitioners. We demonstrate that the intensity of ASCs evoked by HVB was proportional to cardiovascular sympathetic activation and to haemodynamic alterations in cerebral perfusion within clusters spanning the left operculum/posterior insula and right amygdala/anterior hippocampus; regions implicated in respiratory interoceptive representation and the processing of emotional memories, respectively. These observed regional cerebral effects may underlie pivotal mental experiences that mediate positive therapeutic outcomes of HVB.

## Introduction

High ventilation breathwork (HVB) encompasses contemplative and therapeutic practices, including Conscious Connected Breathing or Holotropic Breathwork, in which a controlled pattern of volitional breathing increases the rate or depth of ventilation and is typically accompanied by evocative music. Despite their distinct historical roots and delivery modalities, these different HVB practices share a purported ability to elicit acute extraordinary alterations in subjective experience that closely resemble the qualia of altered states of consciousness (ASCs) induced by psychedelic substances [1–3]. Converging evidence demonstrates the potential value of psychedelic treatments for specific difficult-to-treat psychiatric and physiological conditions [4–7]. The induction of ASCs is suggested to be critical to the therapeutic action of psychedelic substances [8–10], for which HVB might therefore offer a non-pharmacological alternative, with fewer legal and ethical restrictions to large-scale adoption in clinical treatment. In line with this, the popularity of HVB as a therapeutic tool for psychological distress is rapidly expanding, indexed by an increased number of scientific investigations, see [11,12] for more details.

The therapeutic potential of HVB practices is suggested by a long cultural tradition of use to relieve symptoms of psychological distress [11,13] and by emerging preliminary evidence of clinical efficacy from controlled trials in affective and trauma-related disorders [14]. Prolonged hyperventilation/HVB reportedly elicits a wide range of effects on subjective experience that include emotional and psychedelic-like phenomena (ASCs), which range from panic-like sensations to feelings of awe and dissociative symptoms [11]. The 5D-ASC questionnaire is popularly used in ASC research to retrospectively assess such states [15]. A key dimension of this scale is ‘Oceanic Boundlessness’ (OBN); a term coined by Freud in 1920 [16] which describes a set of related feelings including ‘spiritual experience, insightfulness, blissful state, positively experienced depersonalization, and the experience of unity’ [17]. OBN is considered as a defining aspect of ASCs evoked by psychedelic substances, such as psilocybin. However, the neurobiological mechanisms and subjective experience underlying ASCs induced by HVB have not been studied extensively and remain elusive.

First, we characterised the subjective experience of HVB to capture the nature and intensity of evoked experiential phenomena. From these data, we aimed to assess whether these effects could be reliably reproduced in controlled experimental settings in comparison to a remote – and more ecologically valid – condition. These findings informed the choice of the ASC variable, OBN, which was ultimately selected as the most widely reported experiential phenomena to identify the critical neurobiological effects of HVB.

Then we examined the effects of HVB performed by experienced breathwork practitioners in different experimental settings, and investigated peripheral and central neurophysiological mechanisms underpinning ASCs engendered by HVB. The neurobiological endpoints were selected based on well-characterised neurophysiological effects of hyperventilation (reviewed in [11]). Hyperventilation acutely reduces regional cerebral blood flow (rCBF) through interacting effects of hypocapnia, cerebral alkalosis and hypoxia, resulting in transient perturbation of neurometabolic homeostasis. Further, hyperventilation evokes allostatic changes in action-ready bodily arousal, mediated via the autonomic nervous system via the dominance of sympathetic over parasympathetic drive to the heart and blood vessels. In characterising the neurobiological effects of HVB and associated ASCs, we therefore focused our measurements on two robust indices of neurometabolic and autonomic nervous control: rCBF and heart rate variability (HRV).

To our knowledge, no studies have previously reported the relationship between the intensity of ASCs and changes in rCBF induced by HVB practices. Since we could not base our prediction of the regional specificity of such correlations on previous knowledge, we preferred to adopt a whole brain voxelwise exploratory approach. Also, as observations reported on the effects of psychedelics on autonomic nervous system activity have been contrasting [18,19], we could not make precise predictions on the direction of association between HRV and ASCs.

Our study was designed to address different objectives through three inter-related experiments: (please see the methods for more details), in 1) HVB was conducted over an online video-conferencing platform with a breathwork facilitator (REMOTE setting). The aim was to characterise the subjective response to remote HVB in a home setting, and inform the choice of the ASC domain to be used as subjective endpoint. In 2), we used pseudo-continuous arterial spin labelling (pCASL) magnetic resonance imaging (MRI) of the brain during HVB to identify the relationship between neural haemodynamic effects of HVB and subjective measures (MRI setting). In 3) HVB was performed in a psychophysiology lab to characterise the relationship between the psychophysiological effects of HVB (autonomic alterations, see ‘psychophysiological session 3’ for more information) and subjective measures (LAB setting).

## Materials and methods

### Ethics statement

This research was approved by the Research Governance and Ethics Committee (RGEC) in Brighton and Sussex Medical School (BSMS) as ERA/BSMS9AN4/2/2. Recruitment began on 24/11/2021 and ended on 03/03/2022. Participants provided written informed consent before participating.

### Participants

Physically and psychiatrically healthy participants aged 18–65 years old were recruited from the local area through advertisements and flyers distributed at breathwork events, in yoga/meditation centres, and on social media. Participants were eligible to take part if they had either 10 or more experiences of fast-paced breathwork or at least 6 months of any HVB practice (as defined in [11]). They were screened online for eligibility and excluded if they were pregnant, currently experiencing any psychiatric condition (assessed using the Mini International Neuropsychiatric Interview (MINI) to ensure absence of DSM-V listed disorders) [20]. Additional exclusion criteria included a history of epileptic seizures, panic disorder, or syncope in prior HVB, current neurological, musculoskeletal, respiratory, or cardiovascular disease, current pharmacological treatment, and contraindications to MRI.

Participants and were financially compensated £10 per hour for their time and up to £10 in travel expenses.

### Breathwork modality and subjective measures

HVB consisted of cyclic breathing without pausing, accompanied by progressively evocative music. This aimed to reproduce the experience of Conscious Connected Breathing, a widely adopted HVB practice typically led by a trained breathwork facilitator. In the online experimental session, instructions were provided by a breathwork facilitator (DB) who hosted the group of online participants. For the LAB and MRI setting, pre-recorded instructions by the same facilitator were delivered to participants. Specific details on breathwork instructions are presented in the supplementary index (S1 Appendix).

### Questionnaires

Self-reported measures were consistent across the three conditions and administered online via Qualtrics (Qualtrics, Provo, UT) [21] within 30 minutes post-HVB. The selected questionnaires assessed: 1) Affect (Positive and Negative Affect Schedule – Expanded Form, PANAS-X [22]); 2) Panic–like symptoms (Panic Symptoms List, PSL [23]), 3) Fear and discomfort (Visual Analogue Scales (VAS) [24]), and 4) Symptoms of ASCs (5-Dimensional Altered States of Consciousness Rating Scale, 5D-ASC [15]).

Pre-session questionnaires included the VAS (scored from “0 – *no fear and discomfort”* to “100 – *complete fear and discomfort”*) [24] and the PANAS-X, which measured affect using 60 items rated on a 5-point Likert scale from “*1 – very slightly or not at all*” to “*5 – extremely*” [25].

Post-session questionnaires included the VAS and PANAS-X again, as well as the 5D-ASC, which retrospectively assessed ASCs using 94 items rated via VAS [17]. Questions and responses were grouped into 3 broad subscales: oceanic boundlessness (OBN) as previously mentioned, visionary restructuralisation (VRS) to examine the effect of the HVB on vision, and Dread of Ego Dissolution (DED) which captures the anxiety-inducing effects of the experience related to the idea of the dissolution of the self [15]. The PSL, a 13-item tool used to evaluate panic symptomatology, required participants to rate each item on a five-point scale from “*0 – not at all*” to “*4 – extremely severe*”.

The PSL and VAS for fear/discomfort were scored based on [24]: a significant panic attack was indicated by an increase in VAS fear by 50 points and 4 or more items rated above mild in the PSL (mild = 2).

### Online experimental session 1 (REMOTE)

The session was conducted remotely via an online video-conferencing platform (Zoom). Preparatory information was sent to participants after screening and enrolment, including the platform link, instructions, and pre- and post-session questionnaires.

Participants received instructions on camera setup and were assigned personal participant numbers. They were instructed to be in a private room to avoid interruptions and to position their cameras to capture their chest and stomach for observation of full-body, continuous breaths. A breathwork facilitator remotely guided groups of participants through the 30-minute online breathwork session. Members of the research team were present online to ensure session consistency and to monitor participant engagement in HVB practice. No group experience-sharing (integration) followed the breathwork sessions to avoid memory and experience merging. Instead, participants self-integrated (processed) their session by completing retrospective questionnaires that explored the qualia of the experience within 30 minutes of concluding the session.

### Neuroimaging experimental session 2 (MRI)

Each participant in the neuroimaging session was scanned on a Siemens Prisma 3T magnetic resonance imaging (MRI) scanner fitted with a 32-channel head coil. High-resolution structural scans were obtained during rest using a 3D T1-weighted magnetization-prepared rapid acquisition gradient echo (T1 MPRAGE) sequence (repetition time (TR) = 2300 ms, echo time (TE) = 2.19 ms, flip angle = 9°, matrix = 256 × 256, voxel size = 1.0 × 1.0 × 1.0 mm3, GRAPPA acceleration factor = 2; total acquisition time (TA) = 5m 30s). Additionally, twenty control and label images were acquired per condition using a pCASL with background suppression following the parameter recommendation of the consensus paper [26]: label duration = 2000ms, post-labelling delay (PLD) = 1800ms, TR = 5000 ms, TE = 14 ms, voxel size = 3.4 × 3.4 × 6.0 mm3, TA = 3m 22s), was acquired. A proton density (M_0_) image was acquired using the same readout and TR as the control and label images to estimate pcASL CBF.

**Fig 1:**
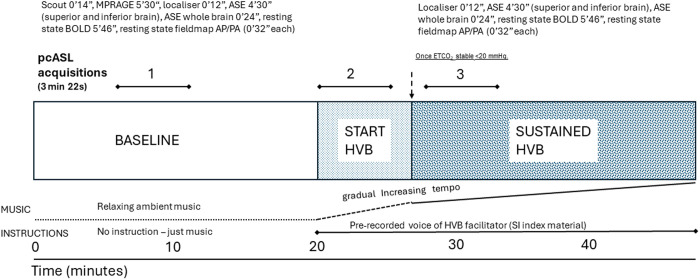
MRI experimental design. pcASL = pseudo-continuous arterial spin labelling, displayed in bold as the primary imaging focus. MPRAGE = 3D magnetization-prepared rapid gradient-echo, ASE = asymmetric spin-echo spiral, BOLD = blood oxygen level dependent, AP/PA anterior-posterior, posterior-anterior. BASELINE = 25 min 32s, breathwork = 23 min 10s. Relaxing music was played at BASELINE during rest for the participant, and evocative up-tempo music that features in breathwork sessions with instructions recorded by a breathwork facilitator were played during HVB.

Fig 1 is a schematic presentation of the MRI experimental design. Recorded instructions (see S1 Appendix) guided participants to breathe through three phases: BASELINE, START, and SUSTAINED HVB. BASELINE consisted of breathing at a normal rate for 20 minutes. Participants were asked to gradually increase their ventilation rate and/or depth via recorded instructions (START HVB) for approximately 6 minutes. The SUSTAINED HVB phase started after at least 5 minutes of START HVB and once end-tidal CO_2_ (EtCO_2_), measured via nasal cannula connected to a capnograph (MICROCAP®, Oridion Medical LTD), was stable at levels ≤20 mmHg (from a typical value of 35–40 mmHg), and was maintained for a further ~20-minutes.

Scans were performed during BASELINE (10 minutes after the scan start), at the start of HVB (immediately after initiating HVB), and during SUSTAINED HVB (after at least 6 minutes of uninterrupted breathwork).

SPM12 [27] and custom written Matlab (The MathWorks Inc., Natick, Massachusetts) scripts were used for image processing and statistics. Rigid-body motion correction was used to realign labelled and control images, and perfusion weighted images were calculated for the realigned control-labelled pairs by subtracting the labelled image from the control. The mean perfusion weighted images were then used to estimate CBF using the one-compartment model [26] and processed to obtain partial volume-corrected [28], tissue-specific (i.e., grey matter (GM) and white matter (WM)) CBF images independently. The pcASL CBF images were normalized maps in MNI space using SPM12 [27]. Participants with prominent arterial transit artifacts in the CBF images, evident by the presence of strong arterial signals upon visual inspection, were excluded from the study.

### Psychophysiological session 3 (LAB)

Participants in the psychophysiology study attended the psychophysiology lab at the Trafford Centre for Medical Research at BSMS. To measure beat-to-beat heart rate (HR) and HRV (computed as the root mean square of successive differences, RMSSD) [29], an electrocardiograph (Firstbeat Bodyguard 2 device) was applied to the torso [28]. To measure EtCO_2_, the participant was fitted with a nasal cannula connected to a capnograph (MICROCAP®, Oridion Medical LTD). Following a BASELINE period during which the participant was instructed to breathe as normal to relaxing music for 10 minutes, the participant was instructed to perform HVB (see supplementary index for more information). This began as a 5-minute warm-up (START HVB) where the participant performed HVB to lower their EtCO_2_ to ≤20 mmHg, followed by 25-minutes of HVB (SUSTAINED HVB) where EtCO_2_ was maintained ≤20 mmHg, aided by recorded instructions from a trained breathwork facilitator. For analyses, the HVB period was defined as starting once EtCO_2_ was stable below 20 mmHg and was subsequently recorded for 20 minutes. The recovery period began immediately after HVB was terminated, and participants resumed their normal breathing. Participants completed retrospective questionnaires within 30 minutes of the session’s conclusion.

#### Data analysis and statistics.

**Objective 1:** To characterize the subjective response to HVB across different settings (REMOTE, LAB, MRI), we compared affective responses (PANAS-X scores clustered into positive and negative domains), panic-like symptoms (VAS fear/discomfort and PSL) and ASCs. For the 5D-ASC, the percentage maximum score was calculated per participant and dimension [30]. Data were compared across the three experiments to test for differences in self-reported effects. A linear mixed-effects model (LMM) was used to examine the effects of setting on the mean responses within each dimension of the 5D-ASC and for the PSL, accounting for subject-specific variability as a random effect using the lmer package in R; [31]. We used the likelihood ratio test to compare a full model with a fixed effect of setting and a random effect of subject to a reduced model with only a random effect of subject [31]. Post-hoc tests were performed using the emmeans package in R [32], adjusted for multiple comparisons using the Tukey method.

For the PANAS-X and VAS, we used an LMM analysis to examine the effects of HVB (pre- versus post-) on setting and the type of question (positive versus negative affect for PANAS; discomfort versus fear for VAS), accounting for subject-specific variability as a random effect. We explored two-way interactions between all the variables and post-hoc tests using the same methods as the previous analyses [32].

**Objective 2:** In order to test the correlations between HVB-induced ASCs and rCBF effects, two contrasts between HVB time points (see MRI experimental design in Fig 1) were studied using paired-samples t-tests in SPM12 [27]: contrast 1) between BASELINE and START HVB grey matter CBF; contrast 2) between BASELINE and SUSTAINED HVB grey matter CBF. Following this, voxel-wise maps of changes in pcASL rCBF (ΔrCBF) were calculated for the contrasts between BASELINE versus SUSTAINED HVB, and START versus SUSTAINED HVB. These ΔrCBF maps were then used for voxelwise correlation analysis with the OBN score, selected as the highest-rated 5D-ASC dimension. Voxel clusters were considered significant at a cluster-forming threshold of p < 0.001, corrected for multiple comparisons using family-wise error (FWE) correction, with a significance level of p < 0.05.

**Objective 3:** To investigate changes in cardiac autonomic drive during HVB and their relationship to the intensity of ASCs, we calculated change in HRV (ΔRMSSD) and HR. Heartbeat timing data were imported from the Firstbeat Bodyguard device into Kubios 3.5.0 HRV scientific 4.1.0 for processing. Raw data contained substantial noise from chest movement. After an initial phase of artefact removal, interbeat interval (IBI) data were smoothed using an automatic noise detection filter at a medium threshold [33]. A repeated measures ANCOVA with contrasts for different orders of an equation was conducted using HRV and OBN (5D-ASC selected from objective 1) as covariates to explain HRV variance over time, with polynomial contrast applied to identify linear and complex patterns, allowing us to characterise dynamic trends in HRV responses to HVB.

## Results

Self-reported data from 42 participants were analysed, comprising 31 unique individuals. Study participants enrolled in one to three experimental sessions: n = 15 (age 42.9 ± 12.6 years, 5 females) for the online experimental session; n = 8 (age 41 ± 13.02 years, 2 females) for the session conducted within the psychophysiology lab; and n = 19 (age 43.7 ± 11.9; 7 females) for the MRI experimental session. There were different participants in each condition (3 repeated all conditions, 5 participants participated in 2 conditions, see S1 Fig for key subjective effects of repeated participants). No adverse events, including panic attacks, were reported across all experimental settings.

### Subjective effect results

**Fig 2:**
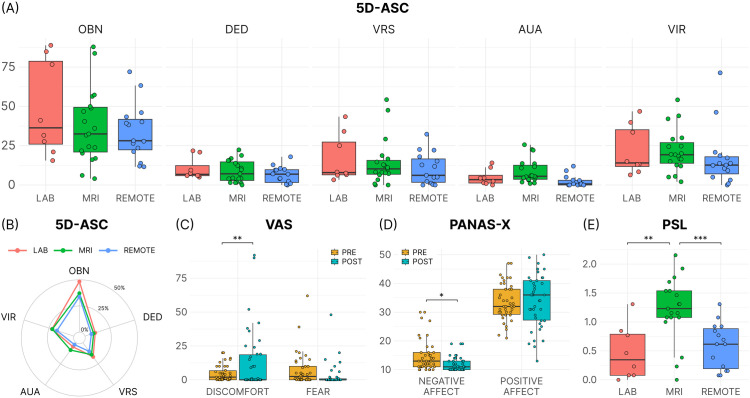
Analysis of subjective effects. A) Averages of the % maximum scores of the 5 dimensions of the 5D-ASC for each of the 3 settings. OBN = oceanic boundlessness; DED = dread of ego dissolution; VRS = visionary restructuralisation; AUA = auditory alterations; VIR = vigilance reduction. **B)** For the 5D-ASC, a linear mixed-effect model did not show an effect of setting (χ2(2) = 3.66, p = 0.160; see ‘Data analysis and statistics’). **C)** Fear and discomfort assessed by VAS indicated no significant effect of experimental setting on HVB, although there was a trend increase in discomfort and reduction in fear after HVB across all environments. **D)** Negative affect assessed by PANAS-X was reduced by HVB. There was no significant effect of HVB on positive affect, and no significant effect of environment on negative or positive affect. **E)** For the PSL, a linear mixed-effect model revealed a significant main effect of setting (χ2(2) = 20.41, p < 0.001; see ‘Data analysis and statistics’). Post-hoc Tukey tests identified significant differences between LAB vs. MRI and MRI vs. REMOTE (LAB – MRI, t(22.11) = −3.78, p = 0.003; LAB – REMOTE, t(28.84) = 0.041, p = 0.999; MRI – REMOTE, t(34.09) = −4.37, p < 0.001).

Generally, measures of fear and discomfort were low in all conditions (Fig 2, panel C). LMM analyses identified only a significant two-way interaction between HVB and question type (fear and discomfort), with post-hoc tests showing a significant increase in discomfort post-HVB. These results indicate that perceived discomfort increased during HVB, while perceived fear remained stable across settings.

None of the mean individual post-HVB PSL scores were ≥ 2, indicating that no panic symptoms reached a clinically significant threshold (a score of 2 corresponded to ‘moderate’). However, the LMM identified a significant main effect of setting on the PSL, with post-hoc analyses showing the PSL scores were significantly higher in the MRI setting compared to the LAB and REMOTE settings (Fig 2, panel E). LMM analyses on the PANAS-X found only a significant two-way interaction between pre/post-HVB and affect, with post-hoc analyses revealing a significant decrease in negative affect from pre- to post-HVB (Fig 2, panel D). These results indicate that HVB decreased negative affect, while positive affect remained stable across settings.

In all three settings, HVB elicited altered subjective experiences indicated by positive scores in all dimensions of the 5D-ASC (Fig 2, panels A and B). The highest rated phenomenon was OBN across all three settings, which was selected as a meaningful proxy indicator of the subjective effects of HVB for analysis. A LMM revealed no significant main effect of setting and no significant interaction effect between setting and dimension (Fig 2, panel B).

### MRI results

Datasets from 13 participants (mean age 43.7 ± 11.9; 7 female) were included in the analysis. Voxel clusters showing statistically significant CBF reductions occupied 12% and 28.5% of global grey matter volume at START and SUSTAINED HVB, respectively. Global CBF was reduced by 30.5% during START (36.2 ± 12.4 ml/100g/min) and 41.6% during SUSTAINED HVB (30.5 ± 8.5 ml/100g/min) relative to BASELINE (52.2 ± 8.2 ml/100g/min).

**Fig 3:**
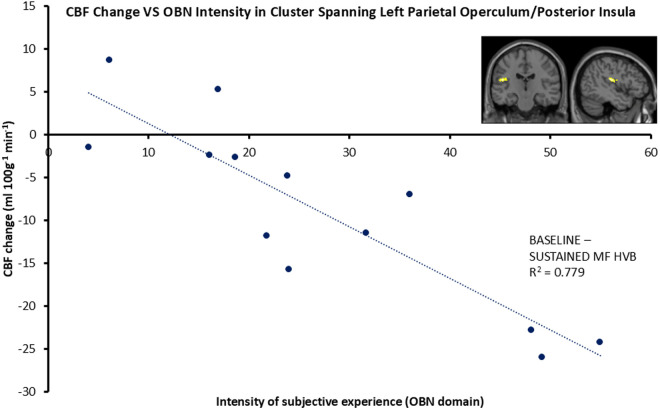
Negative correlation between CBF and intensity of subjective experience (OBN) during BASELINE HVB (p = 0.0475) and SUSTAINED HVB in the left posterior insula/parietal operculum. Data points represent cluster means. Statistical significance was assessed using cluster size inference (initial cluster-forming threshold: p < 0.001; corrected FWE: p < 0.05).

We first identified a region of cortex where HVB-evoked reduction in perfusion (ΔrCBF from BASELINE to SUSTAINED) correlated with the magnitude of ASC in the OBN dimension. This cluster encompassed left parietal operculum/posterior insula (PO/INS) (Fig 3). Here, rCBF in PO/INS was reduced by 7.7% during the START period of HVB (43.7 ± 10 ml/100g/min) and 18.9% during SUSTAINED HVB (38.4 ± 12.7 ml/100g/min) relative to BASELINE (52.2 ± 8.2 ml/100g/min).

**Fig 4:**
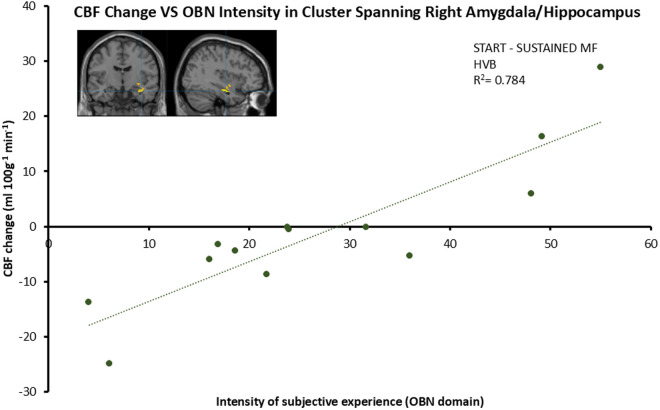
Positive correlation between the intensity of subjective experience (OBN) and ΔCBF in the right basolateral nuclei of the amygdala that extends to CA1 areas of the hippocampus during SUSTAINED HVB, relative to START. Data points represent cluster means. Statistical significance was assessed using cluster size inference (initial cluster-forming threshold: p < 0.001; corrected FWE: p < 0.05).

Next, we identified regions of significant positive correlation between OBN score and ΔCBF changes from START to SUSTAINED (Fig 4). The cluster encompassed right basolateral amygdala and extended to the CA1 region of anterior hippocampus (BLA/CA1) rCBF. Statistical significance was assessed using cluster size inference (initial cluster-forming threshold: p < 0.001; corrected FWE: p < 0.05).

**Fig 5:**
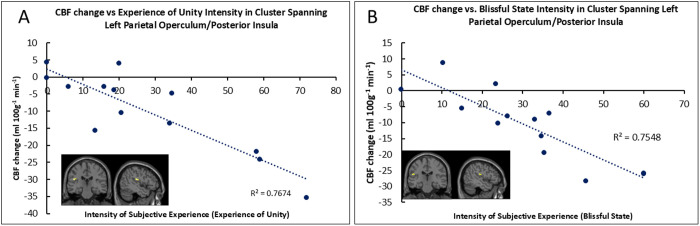
A. Negative correlation between the intensity of subjective experience (experience of unity) and CBF in PO/INS during SUSTAINED HVB, relative to BASELINE, and B. Negative correlation between the intensity of subjective experience (blissful state) and CBF in PO/INS during SUSTAINED HVB, relative to BASELINE. Data points represent cluster means. Statistical significance was assessed using cluster size inference (initial cluster-forming threshold: p < 0.001; corrected FWE: p < 0.05).

We then investigated if specific concepts related to OBN, namely, disembodiment, experience of unity and blissful state [17], were associated with ΔCBF in PO/INS from BASELINE to SUSTAINED. Analyses identified negative correlations between ΔCBF and the experience of unity (Fig 5A) and blissful state (Fig 5B), but not disembodiment, indicating that the magnitude of subjective experience related to bliss and unity correlated with a reduction in perfusion in this region.

### HRV results

**Fig 6:**
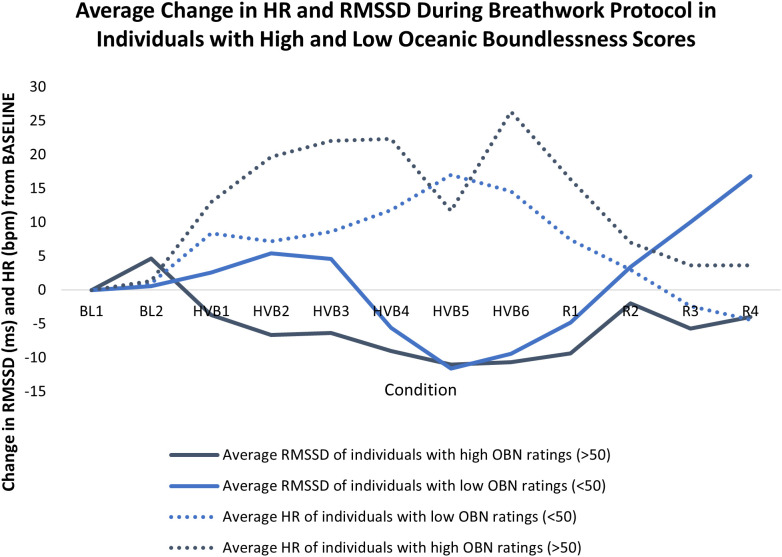
The exploratory mean change from BASELINE in RMSSD (ms) and HR (bpm) across participants during the breathwork protocol after the first BASELINE period (of 5 minutes) in individuals with high (n = 3) and low (n = 5) oceanic boundlessness (OBN) scores. BL = baseline, HVB = high ventilation breathwork, R = recovery. Each period = 5 minutes.

A repeated-measures ANCOVA demonstrated an effect of time on RMSSD through BASELINE and phases of HVB and recovery, indicating a significant within-participant cubic effect. There was a significant interaction effect between OBN and the polynomial cubic contrast, F(1, 6) = 6.211, p < 0.05 (Fig 6).

Participants were classified into high and low OBN responders using a cutoff score of 50 (50% of the maximum score) to identify ASC experience based on criteria in psychedelic drug literature, e.g., [34].

## Discussion

Our study is the first that aims to characterise the relationship between the intensity of ASCs induced by HVB practices and associated central and autonomic nervous system effects, via changes in pcASL rCBF and HRV, respectively. Our findings identified significant correlations that might indicate possible neurobiological substrates of HVB-evoked ASCs, accompanied by music. First, our findings demonstrated that the experience of HVB can be safely reproduced across different experimental settings. The pattern of self-reported experiences elicited within the MRI scanner and psychophysiology lab were analogous to those experienced at home (a more natural and comfortable environment).

During all experimental sessions, participants reported a trend-level reduction in ratings of fear and negative emotions. Additionally, no adverse reactions or panic attacks were experienced by any participants. However, there was a mild increase in discomfort ratings and mild panic-like symptoms were reported during the MRI session – although these were not associated to increased anxiety or panic attacks and might have been evoked by the physical constraints of being inside the MRI chamber. This experience conflicts typical breathwork practice. The observed increase in physical discomfort alongside reductions in fear and negative affect may reflect a hormetic effect, whereby transient physiological or emotional stressors promote longer-term psychological resilience [35,36].

Across participants and experimental settings, HVB reliably enhanced ASCs dominated by OBN, which is considered as a defining aspect of ASCs evoked by psychedelic substances, such as psilocybin. Although differences in OBN scores across conditions were not statistically significant, participants in the LAB condition reported noticeably higher scores compared to MRI and REMOTE sessions. This trend suggests that the in-person experience and controlled, quiet environment of the LAB may have enhanced the immersive and evocative qualities of the breathwork experience. In contrast, the MRI setting—despite also involving in-person contact —may have been less conducive due to scanner noise and physical constraints. Finally, the REMOTE condition, which involved no in-person interaction and took place in participants varied home environments, received the lowest OBN ratings. These findings point to the importance of ‘set and setting’, e.g., [37], a concept well-established in psychedelic research in shaping ASCs and subjective response. These results may indicate that physical context and interpersonal presence play a critical role in breathwork outcomes.

Interestingly, OBN is reportedly the most accurate predictor of antidepressant actions of psychedelic substances [9,38–40]. In the present study, the magnitude of the OBN experience induced by HVB was comparable to that elicited by serotonergic psychedelic substances, including psilocybin and lysergic acid diethylamide (LSD) [41,42], reinforcing observations of other breathwork studies [1,2].

Fast-paced hyperventilation induces cardiovascular sympathetic activation (with relative cardiovagal parasympathetic withdrawal) and cerebral vasoconstriction, the latter is a consequence of respiratory alkalosis, resulting from reduced plasma CO_2_ and H^+^, which elevates blood pH [43]. Ergo, we focused our analysis on correlations between OBN and changes in HRV (for which decreases capture pro-sympathetic shifts in cardiac autonomic balance) and CBF (where regional reductions reflect cerebrovascular vasoconstriction). Importantly, decreased EtCO_2_ is implicated as a critical factor in catalysing stronger, deeper ASCs during breathwork and may predict subacute psychological and physiological outcomes [2,44].

Our experiments identified that HVB engendered substantive time-dependent decreases in pcASL CBF. The reduction of rCBF relative to BASELINE in a cluster localized within left posterior insula and left parietal operculum predicted the intensity of OBN. This region encompasses primary interoceptive cortex and represents the state of cardiorespiratory arousal, and is consequently engaged in higher-order respiratory control. Correspondingly, neural activity here is enhanced by chemo-stimulated increases in ventilation [45]. More broadly, this insular-parietal region supports the integration of somatosensory information as well as the cortical representation of afferent respiratory and bodily signals [46], for example in the emergence of conscious motor intentions [47]. It is also implicated in high-level integrative processes which merge external and internal stimuli [48,49] that contribute to a coherent, embodied representation of self [50].

These roles are relevant to the interpretations of our finding that rCBF reductions within the PO/INS during SUSTAINED HVB predicted the intensity of self-reported OBN experience. The construct of OBN is closely related to feelings of depersonalization, sense of unity and blissful state [16], and our findings align with the notion that abnormal integration of signals in the posterior insula cortex can result in abnormal body ownership. Studies on ecstatic epilepsy, a rare focal epilepsy, show that the insula is involved in ASC-like experiences, including “heightened self-awareness, mental clarity and unity with everything that exists, accompanied by a sense of bliss and physical well-being” [51]. These overlap with the OBN construct, as assessed in the 5D-ASC [15]. Picard and Craig [52] hypothesised the involvement of the insula in the genesis of ecstatic epilepsy given its role in interoception and self-consciousness, confirmed by intracerebral electrode recordings [53]. Seizures in the mesiotemporal region propagated to the dorsal anterior insula coincide with ecstatic symptoms [54] and stimulation of the mid-dorsal insula region evoked experiences akin to oceanic psychedelic effects [55]. The interruption of interoceptive predictive coding during anterior insula seizures may underlie the ecstatic experience, emphasizing its pivotal role in mystical and psychedelic states [51,52].

We then ran an exploratory subsequent analysis (Fig 5) to explore the correlations between sub-dimensions of the 11D-ASC relevant to OBN [17], namely ‘disembodiment’, ‘unity’ and ‘bliss’, and CBF reductions induced by HVB relative to BASELINE. We found that scores of ‘unity’ and ‘bliss’, but not ‘disembodiment’ were associated with CBF reductions (see S1-S3 Tables for details), which concurs with the notion discussed above, specifically pointing to an involvement of the insula in the genesis of blissful feelings. Of note, 5-HT2A– targeting compounds such as psilocybin, renowned for therapeutic utility, are known to induce CBF decreases in different brain regions that correlate with the intensity of psychedelic experiences [56]. Studies found reduced CBF in the insula following psilocybin administration, though it was not linked to the subjective effects assessed via the 5D-ASC [57].

CBF reductions occur rapidly following decreased EtCO_2_ and can be reliably detected in response to very brief periods of hyperventilation. Conversely, the profound experiential changes in emotion and thought processes tend to emerge gradually, and correspondingly psychedelic phenomenology appears contingent on the length of the breathwork session [58]. Our investigation therefore compared HVB-evoked effects during late (SUSTAINED) and early (START) phases of HVB to focus specifically on the haemodynamic changes that occurred once the subjective effects had emerged. By contrasting the START and SUSTAINED phases of hyperventilation, we identified a region of right amygdala/anterior hippocampus, where the intensity of subjective experience (OBN as a covariate) positively correlated with CBF changes. Here, increased rCBF predicted the most intense OBN experience, despite the global reduction in CBF. One possible explanation is that this amygdalo-hippocampal CBF increase reflected increased regional neural activation associated with emergent expression of intense subjective effects. These regions are well established as being specialized for emotion (amygdala) and memory (hippocampus) processing. Their reciprocal interactions enable the formation of episodic representations, integrating the emotional significance and interpretation of memories [59]. Interestingly, increased right amygdala blood-oxygen level-dependent responses to emotional faces in functional MRI (fMRI) have been identified in patients with treatment-resistant depression following administration of psilocybin, predictive of clinical improvements at 1 week. The authors posit that this indicates psilocybin aids individuals’ ability to confront, reappraise, and improve emotional responsiveness [60]. Though speculative, these findings may indicate that HVB facilitates the processing of emotionally salient memories, which is proposed to be an essential therapeutic element in psychedelic-assisted psychotherapies [61,62], consistent with promising results from clinical trials of HVB applied as a therapy for patients with post-traumatic stress disorder (PTSD) [63]. Our finding of increased CBF in the hippocampal region also indicates that a single session of intense hyperventilation selectively alters perfusion to the hippocampus, similar to the effects observed after 20 minutes of hypocapnia-inducing moderate exercise [64]. This finding was interpreted as indicative of short-term metabolic adaptation to the high energy demands of hippocampal neurons, rather than reflecting the effects of mechanical vascular changes [64].

HRV is a physiological index of the balance of sympathetic and parasympathetic influences on the heart. The observed reduction in HRV reflects sympathetic activation and withdrawal of parasympathetic drive [65–67], consistent with an action-ready state of psychophysiological engagement. As previously described, OBN characteristically entails a deeply felt positive mood linked to the experience of unity with the self and the world, and in its extreme, is experienced as a mystical or religious experience [17]. Our finding that OBN ratings were related to changes in HRV over time echoes observations from other relevant modalities of ASC induction, such as self-induced cognitive trance [68] or 5-HT agonists, such as LSD [18,69–71] or N,N-Dimethyltryptamine (DMT) [72,73]. Nevertheless, the observed association with a positive mood contrasts the wide acceptance of reduced HRV as an index of stress and negative affective states. During HVB, the physiological state of the body exceeds typical homeostatic boundaries, and the autonomic correlates of this may reinforce a dissociative psychological state of positive affect in line with the premise of hormesis, see [11] for more.

This exploratory work is preliminary and limited by a small sample size and a lack of an independent control condition that may, in our neuroimaging session, have prevented us from separating the contributions of haemodynamic effects secondary to CO_2_ changes from neural activation. We also acknowledge the possibility that our whole brain voxelwise approach has led to reduced sensitivity to detect significant correlations relative to an a priori region of interest (ROI) approach. In addition, from a methodological perspective, it may be argued that the lack of control group exposed only to music may prevent dissociation of the effects of auditory stimuli (the ambient track) and the effects of HVB. Listening to upbeat music does stimulate ventilation and this should be controlled in the future. Helpful input from lived experience populations indicates that music is a powerful support to therapeutic applications of HVB. In choosing our design, we elected not to “dissect” the music component from HVB, as we preferred to consider HVB as a contemplative practice accompanied by music as a whole – and to test it in its entirety [74]. Importantly, both studies [2,75] have dissected the effect of music and breathwork, and elucidate that music is not the main factor in triggering ASCs. This reveals that music alone is unlikely to engender significant ASCs of the magnitude reported in this research. It is also argued that the reductionist approach of breaking down a contemplative practice to dissect an “active ingredient” is less preferable than testing a practice in its entirety [74]. Conjointly, psychedelic therapy sessions do not adhere to one specific psychotherapeutic model, though one consistent feature of the experience is listening to music. During psychedelic therapy sessions, patients are urged to centre their attention inwardly whilst supine and listening to a carefully selected playlist. It is suggested that music can help facilitate therapeutic experiences. As such, we are looking for CBF changes that correspond with ASCs, induced by HVB with music. We elected to proceed to treat the experience as whole, hence no control for music. More hypothesis-driven work is certainly required within the neuroscience of breathwork.

Furthermore, our study focused on experienced HVB practitioners and therefore our results may not be generalisable; though this is also a strength as it ensured our practitioners were able to reach the desired state without adverse outcomes. Additionally, we were unable to directly assess relevant mechanisms of therapeutic actions, as our subjective measures did not include clinical parameters relevant to traumatic memories or other forms of psychological distress. Moreover, ASCs initiated by breathwork are inherently dynamic, thus dynamic phenomenological tools, such as those employed by Lewis-Healey et al., [58] may be more sensitive and informative.

Our imaging approach was also limited by the absence of correction for physiological noise caused by cardiac and respiratory fluctuations. This is crucial for most physiological fMRI applications for reducing signal artifacts. However, physiological fluctuations (including respiratory frequency and volume changes) can still introduce signal variations in ASL data, but we believe that our approach remains robust. Our analysis inherently averages over multiple cardiac cycles, which helps mitigate the effects of physiological noise on CBF quantification, and although respiration-induced signal changes are observed in some pcASL studies, they are largely associated with motion rather than direct vascular effects [76]. This does not fully eliminate the influence of an overall increase in HR, respiratory rate, or other physiological parameters during changes in breathing conditions—factors that may also impact labelling efficiency and arterial transit times. These considerations are relevant not only to our study, but to other ASL studies where complex, interconnected factors can influence ASL signal and CBF. Practical constraints limit the extent to which additional physiological measurements can be incorporated, particularly in a study as complex as ours. These constraints include scanning time and the potential stress on participants. Furthermore, measuring CBF at the start of the SUSTAINED HVB rather than at the end or during is not ideal, yet, it may capture the physiological ‘trigger condition’ for entry into ASCs, which can continue irrespective of specific physiological threshold conditions, see [2] for more.

A further key reason for choosing not to adopt a method of correction for EtCO_2_ was a high risk of regressing out our primary signal of interest. One plausible mechanistic hypothesis inspiring our work is that ASCs evoked by HVB practices directly result from alterations in EtCO_2_ and the resulting cerebral pH, causing downstream effects on neuronal function [11]. Therefore, CO_2_ related physiological signals are likely correlated with experimental effects that were the focus of our investigation, in line with recent work by others [2] and regressing out these signals would severely impact our sensitivity to study the relationship between haemodynamic effects and ASCs. An additional reason is that our measures of EtCO_2_ (collected by nasal cannula and capnograph) have been occasionally incomplete during the end of HVB sessions when EtCO_2_ levels had reached particularly low levels, and we felt that application of correction methods using incomplete physiological data would have risked introducing major artifacts.

Additionally, we observed prominent arterial transit time (ATT) artifacts in 6 out of 19 participants. These artifacts were characterized by strong arterial signals in the CBF images, which led to their exclusion from the analysis. ATT artifacts can significantly impact the accuracy of perfusion measurements, as they indicate that labelled blood has not yet reached the capillary beds. To address this issue in future research, capturing blood flow dynamics at different delay times through a multi-PLD ASL protocol would reduce the impact of ATT artifacts and increase precision of assessment of cerebral perfusion.

## Conclusion

In conclusion, our exploratory experiments suggest that circuitries supporting the integration of interoceptive representations and processing of affective memories are putative neurobiological substrates of HVB-induced ASCs. Our findings indicate directions for future research towards a better understanding of HVB and ultimately harnessing such practices for future therapeutic applications.

## Supporting information

S1 Appendix Breathwork instructions. (DOCX)

S1 Table Coordinates of significant clusters observed when correlating the intensity of subjective experience (OBN) with ΔCBF during contrasts: BASELINE versus SUSTAINED and START versus SUSTAINED. (DOCX)

S2 Table Coordinates of significant clusters observed when correlating the intensity of subjective experience (Experience of Unity, a component of OBN/5D-ASC) with ΔCBF during BASELINE to SUSTAINED. (DOCX)

S3 Table Coordinates of significant clusters observed when correlating the intensity of subjective experience (Blissful State, a component of OBN/5D-ASC) with CBF during BASELINE to SUSTAINED. (DOCX)

S1 Fig Graph of key subjective effects for repeated participants. (DOCX)
