# Supplementary material for: Neurobiological substrates of altered states of consciousness induced by high ventilation breathwork accompanied by music
Source: PLoS One. 2025 Aug 27;20(8):e0329411. doi: 10.1371/journal.pone.0329411 (PMC12385377; doi:10.1371/journal.pone.0329411)
Supplement: S1 Appendix — (DOCX) [file pone.0329411.s001.docx]

## **S1 Appendix. Breathwork instructions**

Participants were guided through pre-recorded audio instructions accompanied with evocative ambient music played through a speaker in the lab to breathe normally for 10 minutes (baseline) then engage in HVB, encouraged by the tempo of the music progressively increasing to the end of HVB. Some examples of the recorded instructions are presented below.

“Mouth wide open, pulling on the inhale, that’s it. No pauses at the top of the inhale, or the bottom of the exhale. Full body breaths. Breathing in to your whole body. Keep breathing. Getting comfortable, finding your rhythm. Keep going. As you’re breathing, it’s now time to let go of any intention you have, of any expectations you have, just focusing on the breath. Keep going. Active inhale, passive exhale. The music is going to keep on rising, so fall into the rhythm and let your breath guide you. Your job is just to keep breathing, pulling on that inhale. Surrendering to the exhale. Keep that breathing circular, that’s it. Keep going. Whatever sensations you’re feeling, let them come, let them rise, enjoy them. Stay focused. Give yourself fully to the breath. It’s your closest friend. It will be with you from the moment of your birth and stay by your side until you die. You can trust it.”
