# Supplementary material for: Neurobiological substrates of altered states of consciousness induced by high ventilation breathwork accompanied by music
Source: PLoS One. 2025 Aug 27;20(8):e0329411. doi: 10.1371/journal.pone.0329411 (PMC12385377; doi:10.1371/journal.pone.0329411)
Supplement: S1 Table — (DOCX) [file pone.0329411.s002.docx]

**S1 Table. Coordinates of significant clusters observed when correlating the intensity of subjective experience (OBN) with ΔCBF during contrasts: BASELINE vs SUSTAINED and START vs SUSTAINED.**

| **Region Name, L/R** | **MNI coordinates [x y z]** | **T-values** | **Cluster** | **Contrast** |
| --- | --- | --- | --- | --- |
| Amygdala_R (aal3v1) | [28 -4 -20] | 7.68 | 170 | START VS SUSTAINED |
| Hippocampus_R (aal3v1) | [36 -6 -22] | 5.86 | 170 | START VS SUSTAINED |
| Hippocampus_R (aal3v1) | [38 -14 -20] | 5.44 | 170 | START VS SUSTAINED |
| Rolandic_Oper_L (aal3v1) | [-48 -20 18] | 6.05 | 121 | BASELINE VS SUSTAINED |
| Insular_L (aal3v1) | [-34 -26 22] | 5.12 | 121 | BASELINE VS SUSTAINED |
| Rolandic_Oper_L (aal3v1) | [-42 -8 16] | 4.59 | 121 | BASELINE VS SUSTAINED |

Statistical significance was determined using cluster size inference with an initial cluster forming threshold of p < 0.001, where clusters with a corrected FWE of p < 0.05 were considered significant. Anatomical information was derived using the xjView toolbox (<http://www.alivelearn.net/xjview>; based on the WFU_PickAtlas, <http://fmri.wfubmc.edu/software/PickAtlas>). In the table﻿ “aal” denotes Automated Anatomical Labeling.
