# Supplementary material for: Neurobiological substrates of altered states of consciousness induced by high ventilation breathwork accompanied by music
Source: PLoS One. 2025 Aug 27;20(8):e0329411. doi: 10.1371/journal.pone.0329411 (PMC12385377; doi:10.1371/journal.pone.0329411)
Supplement: S3 Table — (DOCX) [file pone.0329411.s004.docx]

**S3 Table. Coordinates of significant clusters observed when correlating the intensity of subjective experience (Blissful State, a component of OBN/5D-ASC) with ΔCBF during BASELINE to SUSTAINED.**

| **Region name, L/R** | **MNI coordinates [x y z]** | **T-values** | **Cluster** | **Contrast** |
| --- | --- | --- | --- | --- |
| Postcentral_L (aal3v1) | [-52 -16 22] | 5.46 | 34 | BASELINE VS SUSTAINED |
| Rolandic_Oper_L (aal3v1) | [-50 -24 22] | 5.07 | 34 | BASELINE VS SUSTAINED |

Statistical significance was determined using cluster size inference with an initial cluster forming threshold of p < 0.001, where clusters with a corrected FWE of p < 0.05 were considered significant. Anatomical information was derived using the xjView toolbox (<http://www.alivelearn.net/xjview>; based on the WFU_PickAtlas, <http://fmri.wfubmc.edu/software/PickAtlas>). In the table﻿ “aal” denotes Automated Anatomical Labeling.
