# Supplementary material for: Neurobiological substrates of altered states of consciousness induced by high ventilation breathwork accompanied by music
Source: PLoS One. 2025 Aug 27;20(8):e0329411. doi: 10.1371/journal.pone.0329411 (PMC12385377; doi:10.1371/journal.pone.0329411)
Supplement: S1 Fig — (DOCX) [file pone.0329411.s005.docx]

# **Supplementary Information Appendix**

## **S1 Appendix. Breathwork instructions**

Participants were guided through pre-recorded audio instructions accompanied with evocative ambient music played through a speaker in the lab to breathe normally for 10 minutes (baseline) then engage in HVB, encouraged by the tempo of the music progressively increasing to the end of HVB. Some examples of the recorded instructions are presented below.

“Mouth wide open, pulling on the inhale, that’s it. No pauses at the top of the inhale, or the bottom of the exhale. Full body breaths. Breathing in to your whole body. Keep breathing. Getting comfortable, finding your rhythm. Keep going. As you’re breathing, it’s now time to let go of any intention you have, of any expectations you have, just focusing on the breath. Keep going. Active inhale, passive exhale. The music is going to keep on rising, so fall into the rhythm and let your breath guide you. Your job is just to keep breathing, pulling on that inhale. Surrendering to the exhale. Keep that breathing circular, that’s it. Keep going. Whatever sensations you’re feeling, let them come, let them rise, enjoy them. Stay focused. Give yourself fully to the breath. It’s your closest friend. It will be with you from the moment of your birth and stay by your side until you die. You can trust it.”

**S2 Table. Coordinates of significant clusters observed when correlating the intensity of subjective experience (OBN) with ΔCBF during contrasts: BASELINE vs SUSTAINED and START vs SUSTAINED.**

The statistical significance was determined using cluster size inference with an initial cluster forming threshold of p< 0.001, where clusters with FWE- corrected p<0.05 were considered significant. Peak x, y, and z coordinates are given in MNI space with each voxels and anatomical regions. MNI coordinates in the left–right, Anterior–Posterior, and inferior–superior dimensions, respectively.

(Cluster denotes the total number of voxels in a given cluster, potentially combining several regions and local maxima.) Anatomical information was derived using the xjView toolbox (http://www.alivelearn.net/xjview; based on the WFU_PickAtlas, http://fmri.wfubmc.edu/software/PickAtlas). In the table﻿ “aal” denotes Automated Anatomical Labelling.

| **Region Name, L/R** | **MNI coordinates [x y z]** | **T-values** | **Cluster** | **Contrast** |
| --- | --- | --- | --- | --- |
| Amygdala_R (aal3v1) | [28 -4 -20] | 7.68 | 170 | START VS SUSTAINED |
| Hippocampus_R (aal3v1) | [36 -6 -22] | 5.86 | 170 | START VS SUSTAINED |
| Hippocampus_R (aal3v1) | [38 -14 -20] | 5.44 | 170 | START VS SUSTAINED |
| Rolandic_Oper_L (aal3v1) | [-48 -20 18] | 6.05 | 121 | BASELINE VS SUSTAINED |
| Insular_L (aal3v1) | [-34 -26 22] | 5.12 | 121 | BASELINE VS SUSTAINED |
| Rolandic_Oper_L (aal3v1) | [-42 -8 16] | 4.59 | 121 | BASELINE VS SUSTAINED |

**S3 Table. Coordinates of significant clusters observed when correlating the intensity of subjective experience (Experience of Unity, a component of OBN/5D-ASC) with ΔCBF during BASELINE to SUSTAINED.**

| **Region name, L/R** | **MNI coordinates [x y z]** | **T-values** | **Cluster** | **Contrast** |
| --- | --- | --- | --- | --- |
| Rolandic_Oper_L (aal3v1) | [-46 -24 20] | 5.73 | 84 | BASELINE VS SUSTAINED |
| Sub-Gyral | [-36 -28 24] | 5.56 | 84 | BASELINE VS SUSTAINED |
| SupraMarginal_L (aal3v1) | [-54 -22 18] | 4.32 | 84 | BASELINE VS SUSTAINED |

**S4 Table. Coordinates of significant clusters observed when correlating the intensity of subjective experience (Blissful State, a component of OBN/5D-ASC) with ΔCBF during BASELINE to SUSTAINED.**

| **Region name, L/R** | **MNI coordinates [x y z]** | **T-values** | **Cluster** | **Contrast** |
| --- | --- | --- | --- | --- |
| Postcentral_L (aal3v1) | [-52 -16 22] | 5.46 | 34 | BASELINE VS SUSTAINED |
| Rolandic_Oper_L (aal3v1) | [-50 -24 22] | 5.07 | 34 | BASELINE VS SUSTAINED |

Statistical significance was determined using cluster size inference with an initial cluster forming threshold of p < 0.001, where clusters with a corrected FWE of p < 0.05 were considered significant. Anatomical information was derived using the xjView toolbox (<http://www.alivelearn.net/xjview>; based on the WFU_PickAtlas, <http://fmri.wfubmc.edu/software/PickAtlas>). In the table﻿ “aal” denotes Automated Anatomical Labeling.

## **S5 Appendix. Graph of key subjective effects for repeated participants.**


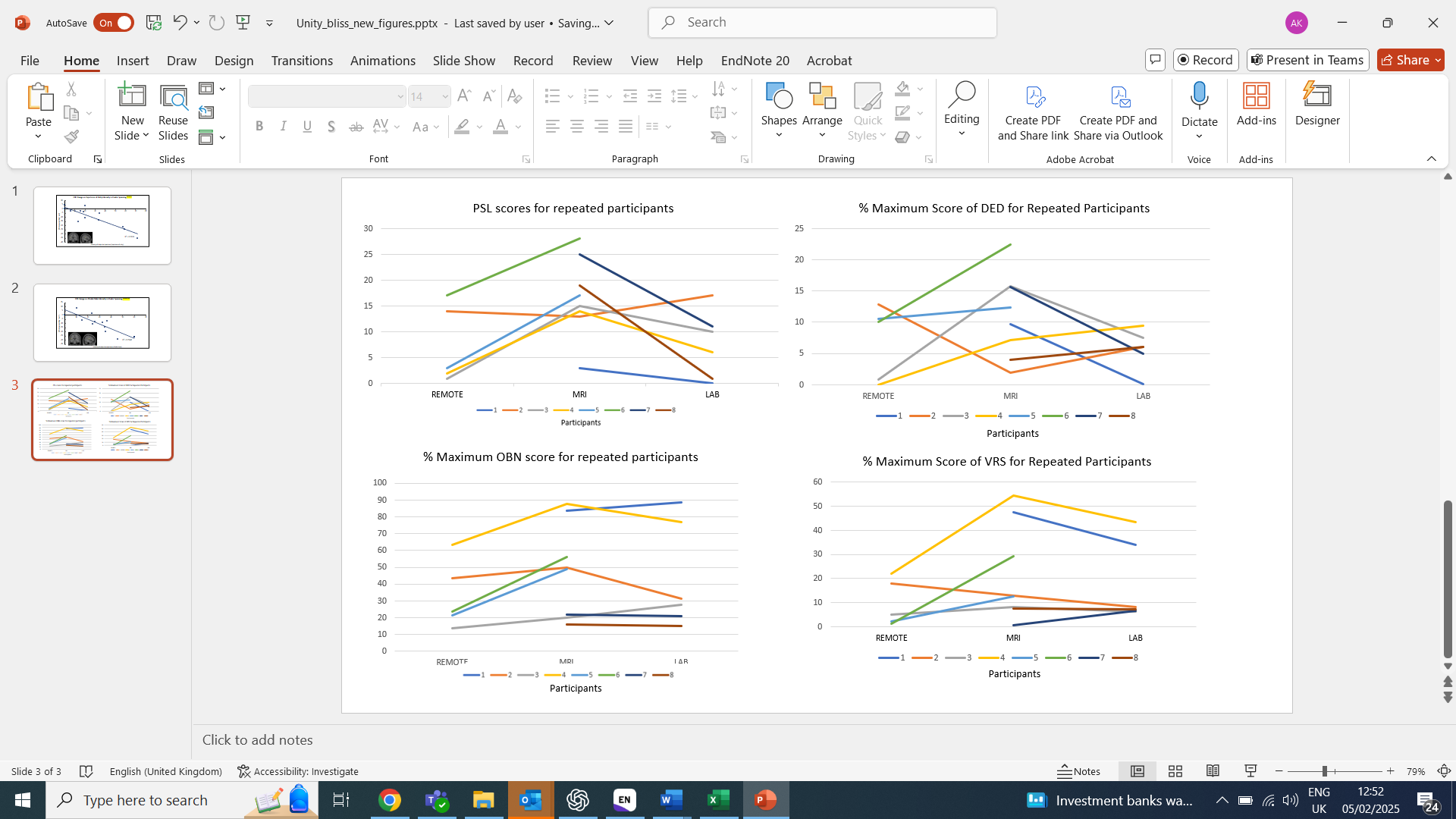


Subjective effects for repeated participants post-breathwork. PSL = panic symptoms list, DED = dread of ego dissolution, OBN = oceanic boundlessness, VRS = visionary restructuralisation.
